# Interventions Associated With Racial and Ethnic Diversity in US Graduate Medical Education: A Scoping Review

**DOI:** 10.1001/jamanetworkopen.2022.49335

**Published:** 2023-01-03

**Authors:** Russyan Mark Mabeza, Briana Christophers, Sophia A. Ederaine, Emily J. Glenn, Zachary P. Benton-Slocum, Jasmine R. Marcelin

**Affiliations:** 1 David Geffen School of Medicine at the University of California, Los Angeles; 2 Weill Cornell/Rockefeller/Sloan Kettering Tri-Institutional MD-PhD Program, New York, New York; 3 Department of Dermatology, University of California Irvine School of Medicine, Irvine; 4 McGoogan Health Sciences Library, University of Nebraska Medical Center, Omaha; 5 Department of Medicine, University of Nebraska Medical Center, Omaha; 6 Division of Infectious Diseases, Department of Internal Medicine, University of Nebraska Medical Center, Omaha

## Abstract

**Importance:**

Racially and ethnically minoritized individuals remain underrepresented in graduate medical education relative to their proportion in the population. While many programs and initiatives have been developed to address this problem, there is little consensus regarding strategies that work to improve representation across specialties.

**Objective:**

To examine and synthesize evidence-based practices that have been used to increase the proportions of underrepresented in medicine (URiM) trainees at US residency and fellowship programs.

**Evidence Review:**

The authors searched PubMed, Google Scholar, Embase, PsycInfo, ERIC, Cochrane Reviews, Cochrane Trials, CINAHL, Scopus, and PROSPERO electronic databases to identify relevant studies published through January 2022. They screened all titles and abstracts for relevance and read full-text articles to identify articles reporting reliable data describing the outcomes of interventions to improve racial and ethnic diversity among trainees.

**Findings:**

Twenty-seven articles were included in this review. Two studies reported on fellowship programs. The most common interventions included holistic review (48%), decreased emphasis on United States Medical Licensing Examination Step 1 scores (48%), and explicit institutional messaging regarding the importance of diversity (37%). A combination of interventions was associated with an increased number of URiM applicants, interviewees, and matriculants across various medical and surgical specialties.

**Conclusions and Relevance:**

In this scoping review, approaches and interventions associated with increased diversity in residency and fellowship programs were identified. Continued efforts are necessary to sustain such efforts and assess long-term outcomes.

## Key Points

**Question:**

What strategies are associated with increased racial and ethnic diversity in graduate medical education programs?

**Findings:**

This scoping review of 27 articles found that combinations of interventions were associated with increased numbers of applicants, interviewees, and matriculants who are underrepresented in medicine across various medical and surgical specialties. Such interventions included holistic review, decreased emphasis on US Medical Licensing Examination Step 1 scores, changes to selection committees, and explicit institutional messaging regarding the importance of diversity.

**Meaning:**

These findings suggest that effective approaches and interventions to increase racial and ethnic diversity in residency and fellowship programs exist and such measures may be beneficial in other graduate medical education contexts.

## Teaser

This scoping review examines and synthesizes evidence-based practices that have been used to increase the proportions of underrepresented in medicine (URiM) trainees at US residency and fellowship programs.

## Introduction

Racially and ethnically minoritized individuals have been historically excluded and remain underrepresented throughout all stages of medicine relative to their numbers in the general population.^1,2^ At the graduate medical education (GME) level (ie, residency and fellowship), individuals from backgrounds underrepresented in medicine (URiM, including Black or African American, Hispanic or Latinx, American Indian or Alaska Native, Native Hawaiian or Pacific Islander, and Southeast Asian individuals) are less likely to receive interviews and be admitted into residency programs.^3,4,5^ Reasons for this discrepancy include low faculty diversity, a lack of URiM students applying, and the inability to match URiM candidates who are highly ranked by residency programs.^6^

Gonzaga and colleagues^7^ provided a framework of possible interventions to diversify the residency class at various stages of the residency application process. A national survey of internal medicine program directors reported multifaceted strategies with a predominance of explicit demonstration of diversity (eg, websites) and fewer resource-intensive approaches, such as URiM-specific events.^8^ While this work, along with a handful of strong perspective pieces and commentaries,^9,10,11,12^ have described what programs should do to diversify their trainee pools, a detailed objective review of such efforts in GME is lacking.

In 2019, the Accreditation Council for Graduate Medical Education (ACGME) revised the common program requirements to require training programs to implement “policies and procedures related to recruitment and retention of minorities underrepresented in medicine and medical leadership,”^13^ and that program evaluations should include assessments of these efforts. However, the ACGME did not provide guidance or best practice recommendations on how programs should meet these requirements. Given the imperative to increase diversity across specialties,^11,14,15,16,17^ this scoping review synthesizes strategies that have been executed to increase racial and ethnic diversity within GME training programs across medical and surgical specialties in the United States.

## Methods

### Literature Search Strategy

**Figure 1.**
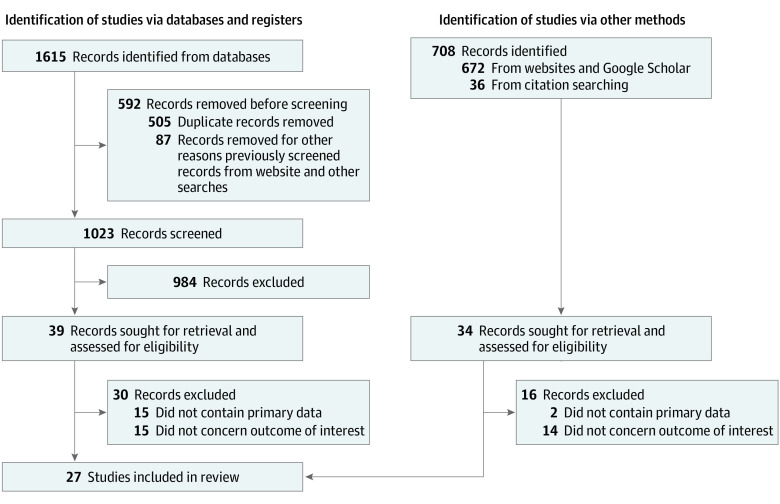
Study Flow Diagram

A protocol for this scoping review was registered with OSF Registries on January 11, 2022.^18^ A librarian/expert searcher (E.J.G.) executed a search of the literature in the following databases: PubMed (1946 to present), Embase (1947 to present), American Psychological Association PsycInfo (EBSCO), ERIC (1966 to present), Cochrane Reviews (Issue 1, January 2022), Cochrane Trials (Issue 12, December 2021), CINAHL (1981 to present), and Scopus (1970 to present). We searched previous studies through Prospero. We used keywords and controlled vocabulary where appropriate to describe *racial and ethnic minorities*, *diversity*, and *recruitment initiatives* concepts for each of the databases searched. The PubMed strategy is included in the eAppendix in Supplement 1. This strategy was translated for other databases. No previously published search strategies were adapted or reused for a substantive part of the search strategies. Results were limited to English language and a date range of January 2011 to January 2022. Results were exported to EndNote, then deduplicated. This study follows the Preferred Reporting Items for Systematic Reviews and Meta-Analyses (PRISMA), specifically for scoping reviews.^19^ The screening workflow is described in the study flow diagram (Figure 1). All literature database searches were conducted on January 17, 2022. In addition to results from the literature search, we searched Google Scholar and reviewed the reference lists of studies that met the inclusion criteria to identify other relevant studies.

### Study Selection

Two reviewers (R.M.M. and B.C.) independently screened the titles and abstracts of studies identified to exclude those that did not meet inclusion criteria. Reviewers read full-text articles to identify articles that included objective data describing the outcomes of interventions on trainee racial and ethnic diversity. Only studies with original data describing concrete interventions to diversify GME training programs were included. Review articles, conference proceedings, meeting abstracts, studies with unclear methodology, and articles inaccessible through the authors’ institutional libraries were excluded. Interrater reliability was assessed using Cohen κ after screening 20 articles. Discrepancies were resolved through reviewer discussion or with the aid of a third reviewer (S.A.E.) as necessary.

### Data Extraction

We extracted data regarding specialty, year of publication, interventions, duration of interventions, and outcomes assessed for each of the studies. Intervention components of interest included medical school clerkships, recruitment prior to residency applications, residency application reviews, interviews, and rank list decisions. Additional outcomes included reporting of postintervention changes in number of URiM applicants, interviewees, ranked candidates, matriculants, and self-reported program attitudes and interests.

One reviewer (R.M.M.) performed initial data extraction, and the second reviewer (B.C.) subsequently extracted data from a randomly selected sample (20%) of the articles to ensure accuracy and consistency. Reviewers compared independent results after each stage of the study selection and data extraction.

## Results

### Study Characteristics

Our search yielded 1615 articles from databases and 708 from websites, Google Scholar, and citation searching (Figure 1). After removal of duplicate articles and abstract screening, 73 full-text articles were reviewed, of which 27 articles published between November 2012 and October 2021 met inclusion in this review.^20,21,22,23,24,25,26,27,28,29,30,31,32,33,34,35,36,37,38,39,40,41,42,43,44,45^ Nineteen studies described data in medical specialties^9,20,21,22,23,27,28,30,31,32,33,35,36,37,38,39,41,43,44^ and 9 in surgical specialties.^21,24,25,26,29,34,40,42,45^ Two studies focused on fellowship programs.^22,43^ Six of the 27 studies were in emergency medicine,^23,27,30,35,38,39^ while family medicine^31,33,44^ and pediatrics^9,37,41^ had 3 articles each. The median duration of the studies was 4 years and ranged between 1 and 10 years. The Table summarizes the major characteristics and outcomes reported in the articles.

**Table.** Description of Study Interventions and Outcomes

Source	Specialty	State	Study type	Study or program size	Study period, y	Study definition of URiM	Intervention details	Outcomes assessed
Deas et al,^21^ 2012	Various specialties within 1 medical institution	South Carolina	Retrospective, observational	500-600 Residents	10	“African American, Latino, Native American”	Development of 8 pathway programs (3 for middle/high school students, 5 undergraduate college students)^a^ MCAT preparation sessions and mini-medical school programs with partner institutions Post-baccalaureate Reapplication Education Program: program for URiM applicants who were not accepted to medical school A Gentleman and a Scholar: mentoring program for high school and college African American males with an interest in health professions Mentoring Assures Medical School Success: monthly roundtable luncheons to dialogue about resources, study skills, and managing various issues Implementation of Second Look Visit program for residency candidates	Overall URiM matriculants to residency increased from 16 (3%) to 36 (7%)^b^ Each specialty enrolled URiM residents aside from dermatology and orthopedic surgery
Auseon et al,^22^ 2013	Cardiology	Ohio	Retrospective, observational	16 Total fellows	5	“African American, Hispanic or Latino, American Indian/Alaska Native, Native Hawaiian/Pacific Islander”	Subcommittee on URiM recruitment Grand rounds–based outreach to diverse internal medicine residency programs Revised interview day agenda to focus on mentorship by including faculty, highlighting available resources, and areas for research and professional growth Targeted postinterview communication with highly competitive URiM applicants	Increased applicants from residency programs with a history of matching URiM medical students: 14 of 483 (3%) URiM applicants in 2011 Matched a URiM fellow for 5 consecutive years
Boatright et al,^23^ 2016	Emergency medicine	National survey	Survey	Data not available based on study design	5	“African Americans, Latinos, Native Americans”	Inclusion of diversity in recruitment materials and institutional website Explicit recognition of diversity in the residency program when URiM applicants arrive to interview Broader selection criteria beyond USMLE scores, including leadership, community service, and life experiences Curricular development on diversity, cultural competence, and implicit bias Pathway program involvement^a^ URiM interview dinners and social events	Program faculty consider applicant URiM status important (OR, 4.9) Program faculty consider engagement in pathway activities (OR, 4.8) and extracurricular activities (OR, 2.6) important
London et al,^24^ 2016	Orthopedic surgery	Data not available	Retrospective, observational	91 Medical students	6	“Non-White, non-Asian”	1-mo Musculoskeletal surgery requirement (orthopedic, plastic surgery, neurosurgical/spine)	URiM applicant increase from 5 of 48 (10%) to 9 of 43 (21%)
Mason et al,^25^ 2016	Orthopedic surgery	National pathway program^a^	Retrospective, observational	118 Medical students	4	“Black, Latino”	8-wk Summer internship program for medical students	URiM applicants: 15 of 48 program participants (31%) vs 783 of 25 676 (3%) of national applicants (OR, 14.5) Black applicants: 12 of 42 program participants (29%) vs 397 of 12 519 (3%) nationally (OR, 15.9) Latino applicants: 3 of 6 program participants (50%) vs 385 of 12 372 (3%) nationally (OR, 32.1)
Nellis et al,^26^ 2016	Otolaryngology	Maryland	Retrospective, observational	15 Medical students	7	“African American, Hispanic, Native American”	2 Visiting clerkship programs (clinical and research)	6 of 7 Clerkship participants who applied to otolaryngology matched
Tunson et al,^27^ 2016	Emergency medicine	Colorado	Retrospective, observational	17 Residents per class	1	“African American or Black, Latino/a, Native Hawaiian, Native American, Vietnamese”	Funded externship Funded Second Look event Increased involvement and visibility of URiM faculty	URiM interview invite: 24 of 344 (7.0%) to 58 of 393 (14.8%) URiM interviewee: 14 of 226 (6.2%) to 49 of 279 (17.6%) URiM matriculant: 1 of 18 (5.6%) to 4 of 17 (23.5%)
Aibana et al,^28^ 2019	Internal medicine	Texas	Retrospective, observational	40 Residents per class	2	“American Indian, Alaska Native, Black/African American, Hispanic/Latino, Native Hawaiian, Pacific Islander”	Implementation of holistic review Standardized interviews Program diversity highlight during interview days	URiM applicants reviewed: 180 of 1276 (14.1%) to 183 of 897 (20.4%) URiM interviewees: 60 of 374 (16.0%) to 95 of 388 (24.5%) URiM matriculants: 5 of 40 (12.5%) to 13 of 41 (31.7%)
Bucknor et al,^20^ 2019	Radiology	California^c^	Retrospective, observational	Not specified in the manuscript	3	Not specified in the manuscript	Summer research experience for high school, college, and medical students Single-day outreach events for high school, college, and medical students Shadowing experiences at county hospital Annual unconscious bias training for residency and faculty interviewers Presence of Diversity and Inclusion Committee on the residency selection committee and all faculty search committees Additional review of students nearly selected for an interview with particular focus on URiM students University level all-day diversity and inclusion training programs Supplemental educational opportunities through departmental grand rounds series	Increased number of applicants to summer research internship: 10 to 29 individuals 67% of interviewed women and URiM students noted that the climate around diversity and inclusion led them to rank the program higher
Butler et al,^29^ 2019	General surgery, orthopedic surgery, plastic surgery, urology, otolaryngology, vascular and thoracic surgery–integrated, neurosurgery	Pennsylvania	Retrospective, observational	16 Residents per class	5	“African American, Hispanic/Latino, Native American/Alaska Native/Native Hawaiian, mainland Puerto Rican”	Funded visiting clerkship program Implementation of holistic review Unconscious/implicit bias talks for recruitment faculty Outreach program from URiM residents and faculty	URiM interviewees: 11.2% to 18.8%^d^ URiM matched: 12.1% to 23.5%^d^ Year 4 milestone: URiM matched in each surgical specialty
Garrick et al,^30^ 2019	Emergency medicine	California^c^	Retrospective, observational	46 Residents, 2 ultrasonographic fellows	12	“Black, Hispanic/Latino, American Indian, Pacific Islander, Alaska Native, Native Hawaiian”	Implementation of holistic review No USMLE Step 1 score filter Increased weight of “gestalt” score Creation of diversity committee Launch of diversity week for URiM applicant interviews	URiM graduates: 12% to 27%^d^ Black residents: 6% to 14%^d^ Hispanic/Latinx residents: 5% to 12%^d^
Guh et al,^31^ 2019	Family medicine	Washington	Retrospective, observational	36-40 Residents per class	4	URiM: “Black, Latinx, Native American”; modified URiM: “people of color except for people of Chinese, Korean, and Indian descent”	Revised mission statement Diversity task force Antiracism curriculum Development of system to evaluate progress	URiM matriculants: 10 of 36 (28%) to 27 of 40 (68%) URiM faculty: 1 of 12 (8%) to 6 of 17 (27%)
Spector et al,^32^ 2019	Neurology	North Carolina	Retrospective, observational	738 Residency applicants	1	“Black/African American, American Indian/Alaska Native, Hispanic/Latino, Native Hawaiian/Other Pacific Islanders”	Removal of minimum USMLE Step score	URiM interview invite: 9 of 45 (20%) to 12 of 49 (24.5%)
Wusu et al,^33^ 2019	Family medicine	Massachusetts	Retrospective, observational	6-12 Residents per class	4	“Black, Hispanic, Native American”	Recruitment at family medicine conferences Established position for director of diversity programs Blinded applicant files during interviews Structured interviews Development of recruitment data analysis metrics	URiM applicants: 29 of 218 (13.3%) to 110 of 402 (19.9%) URiM interviewees: 15 of 136 (11%) to 43 of 206 (20.9%) URiM ranked: 14 of 117 (12%) to 40 of 199 (20.1%) URiM matched: 1 of 6 (16.7%) to 3 of 12 (25%)
Gardner et al,^34^ 2020	General surgery	Florida, Georgia, Ohio, and Texas^c^	Retrospective, observational	2742 Residency applicants (across 7 programs)	1	Not specified in the manuscript	Lowering of minimum USMLE Step 1 score Situational judgment tests based on program values administered to applicants	URiM applicants: 66.0% to 74.1%^d^ Increase of racially and ethnically minoritized applicants considered to take situational judgment tests: 43.9% to 52.7%^d^
Goines et al,^35^ 2020	Emergency medicine	Georgia	Retrospective, observational	115 Medical students	10	Not specified in the manuscript	Partnership between PWI and HBCU to allow for shadowing opportunities, career guidance, away rotation guidance, fourth-year scheduling, application assistance, interview guidance	Proportion of HBCU students matching into emergency medicine: 3.01% to 6.65% (OR, 1.10)^d^
Lewis et al,^8^ 2020	Pediatrics	Missouri	Retrospective, observational	72 Total categorical residents	5	“African American, Hispanic, certain Asian subgroups”	Addition of URiM faculty to selection committee Coffee chat with URiM faculty members for invited interviewees Recruitment at the Student National Medical Association conference 1-mo Funded elective on health disparities 5 URiM-specific interview days	Most diverse intern class: URiM, 6 of 24 (25%)
Barceló et al,^36^ 2021	Psychiatry	California^c^	Retrospective, observational	547 Residency applicants	1	Not specified in the manuscript	Implementation of holistic review, which included “1) identifying and devaluing metrics with known bias and limited predictive value for long-term clinical strength (AOA induction, USMLE scores), 2) reimagining and prioritizing personal qualities and professional characteristics that reflect program values, and 3) actively considering applicants in a broader social context”	Increased odds of URiM selection for interview: ORs, 0.35 vs 0.84^d^ Decreased impact of Step 1 on URiM interview selection: ORs, 2.03 vs 24 Increased impact of GHHS membership on URiM interview selection: OR, 1.59 vs 6.5
Escalante et al,^37^ 2021	Pediatrics	District of Columbia	Retrospective, observational	73 Medical students	7	Not specified in the manuscript	Minority Senior Scholarship Program: pathway program^a^ Travel stipend to all interviewees who self-identify as URiM Intern selection subcommittee that reviews applications of URiM interviewees after routine holistic and mission-aligned review Current URiM resident-ambassadors and longitudinal points of contact for URiM applicants during the interview season Pairing of URiM interviewee with at least 1 URiM interviewer or faculty member with interest in promoting diversity Annual Diversity Grand Rounds and reception at the end of the interview season Second Look visits for all interested applicants with travel expenses reimbursed for URiM applicants that fit closely with the program’s mission Diversity dinner series for URiM medical students, residents, fellows, and attendings	73 Participants completed Minority Senior Scholarship Program, with 19 of 73 (26%) matching at the Children’s National Hospital Increased interest in academic pediatrics: 14 of 20 (70%) to 19 of 20 (95%) URiM matriculants: 2 of 40 (5%) to 21 of 41 (51%), more than 3 times the national average of URiM pediatric residents (16%) and 4 times of URiM physicians nationwide (11%)
Sungar et al,^38^ 2021	Emergency medicine	Colorado	Retrospective, observational	8343 Residency applicants	5	“Black/African American, Hispanic/Latino/of Spanish Origin, American Indian/Alaska Native, Native Hawaiian/Pacific Islander”	Implementation of holistic review, including (1) elimination of score contribution of USMLE scores; (2) creation of a mission score, which assesses an applicant’s alignment with the program’s mission; and (3) creation of a perspective score, which captures the unique perspective that is offered by an applicant, such as race and ethnicity, sexual orientation, first-generation college graduate status, underrepresentation within the program, low socioeconomic status, or disadvantaged background	URiM applicants: 202 of 1347 (15%) to 296 of 1608 (18%) URiM interview invites: 53.8 of 394 (13.7%) to 113 of 456 (15.4%) URiM interviewees: 38.8 of 287.2 (13.5%) to 88 of 411 (21.4%) URiM applicants represented in the top 75 through top 200 cut points based on composite score rank Composite score increase for URiM applicants (67.4% to 77.1%) compared with non-URiM (71.3% to 76.0%)
Lall et al,^39^ 2021	Emergency medicine	Georgia	Retrospective, observational	53-61 Total residents	20	“Black/African American, Hispanic/Latinx, Native American, Native Alaskan, Pacific Islander”	Partnership with Morehouse to provide access to Emory emergency medicine faculty and residents through interest group activities Residency fairs Implementation of holistic review Removal of minimum USMLE Step 1 score Core group of diverse faculty interviewers Diversity and Inclusion committee Establishment of group for the advancement and retention of women in emergency medicine leadership	Consistently higher percentage compared to national norms: Women: 34 of 61 (55.7%) vs 2885 of 8029 (35.9%) Black/African American: 16 of 61 (26.2%) vs 382 of 8029 (4.8%) Overall URiM residents: 20 of 61 (32.8%) vs 1077 of 8029 (13.4%)
Llado-Farrulla et al,^40^ 2021	Plastic surgery	Pennsylvania	Retrospective, observational	3 Residents per class	5	“African American, Latino, Native American/Alaska Native”	Funded clerkship program for URiM students with faculty and resident mentorship Implementation of holistic review Establishment of outreach for URiM candidates through the Alliance of Minority Physicians	URiM residents: 0% to 29%^d^ Higher URiM representation compared with the national average
Marbin et al,^41^ 2021	Pediatrics	California^c^	Retrospective, observational	28 Residents per class	3	Not specified in the manuscript	Implementation of holistic review Diversity committee reviewing URiM applicant pool for interview invites Elimination of applicant photographs during file review and ranking Unconscious bias training for everyone involved with resident recruitment and selection	URiM matriculants: 11% in 2015 (3 of 28) to 13 of 29 (45%) (OR, 6.8) in 2019 and 10 of 28 (36%) (OR, 4.6) in 2020 Ranking Advising Committee membership increased from 60 to 80, with 25%-30% URiM members
Nehemiah et al,^42^ 2021	General surgery	Pennsylvania	Retrospective, observational	15 Residents per class (8 categorical, 7 preliminary)	3	Not specified in the manuscript	Implementation of holistic review Expansion of interview selection committee to include a diverse group of faculty and residents Blinding of selection committee and interviewers to USMLE scores and grades Emphasis on key characteristics and attributes aligned with department’s mission Implementation of standardized scoring sheet focusing on characteristics	URiM ranked: 14% to 20%^d^ URiM matched: 14% to 21%^d^
Rymer et al,^43^ 2021	Cardiology	North Carolina	Retrospective, observational	8 Fellows per class	3	“Black, Hispanic, Latinx, Native American”	Creation of a diversity and inclusion task force Changes in program leadership Diversification of recruitment committee Removal of minimum USMLE Step score 3 Application reviewers per applicant URiM applications reviewed by URiM faculty group Preinterview dinners Pairing of interviewees with potential research mentors Pairing of interviewees with at least 1 URiM interviewer End-of-day reception with faculty	URiM applicants: 10.5% to 12.5%^d^ URiM interviewees: 14.0% to 20.0%^d^ URiM ranked: 3.7% to 6.7%^d^ URiM matriculants: 5.6% to 33.3%^d^
Stoesser et al,^44^ 2021	Family medicine	Utah	Retrospective, observational	10 Residents per class	4	“Black, Latinx, American Indian/Alaska Native, Pacific Islander, Southeast and refugee Asian”	Funded visiting clerkships for URiM applicants Implementation of holistic review Blinding of reviewers to applicant photos Increased interactions of applicants with URiM faculty and residents during interview day Lower minimum USMLE Step 1 score with only passing score required Outreach calls by the Office of Health Equity, Diversity and Inclusion to individual URiM potential residents who interviewed with the program Panel discussion with current URiM residents for those participating in visiting clerkship	URiM interviewees: increased 16-fold over 5 years, from 2 of 68 (3%) to 33 of 98 (34%) URiM matriculants: 50% in the 2021 match cycle (5 of 10) Latinx residents increased from 0 to 6, and underrepresented Asian residents from 0 to 2
Wallace et al,^45^ 2021	Urology	National pathway program^a^	Retrospective, observational	66 Medical students	1	“African, Caribbean/West Indian, Hispanic/Latinx, Indian, Mixed”	Implementation of the R. Frank Jones Urology Interest Group Educational webinars that include prerequisites and extracurricular requirements necessary to match Application polishing program with mock interviews Research education through webinars, research connections, and paid research opportunities Introduction of students to academic urology faculty volunteers, who serve as sponsors and advocates for URiM students	At the end of 2020, 66 students (60% Black, 21% Latinx) were registered members of the R. Frank Jones Urology Interest Group 31 of 39 Applicant members matched in the 2021 urology match

Abbreviations: GHHS, Gold Humanism Honor Society; HBCU, historically Black college or university; MCAT, Medical College Admission Test; OR, odds ratio; PWI, predominantly White institution; URiM, underrepresented in medicine; USMLE, US Medical Licensing Examination.

^a^ Pathway is used to refer to pipeline programs, in consideration of American Indian and Alaska Native communities who experience the consequences of oil companies transporting crude oil through their sacred lands.

^b^ Interventions and outcomes are reported as consistent with language used in original studies. The term URiM, which captures underrepresented minorities and underrepresented in medicine, as described in the reviewed articles, was standardized.

^c^ State where affirmative action ban is active.

^d^ Raw numbers were not reported in the study manuscript.

### Intervention Characteristics

**Figure 2.**
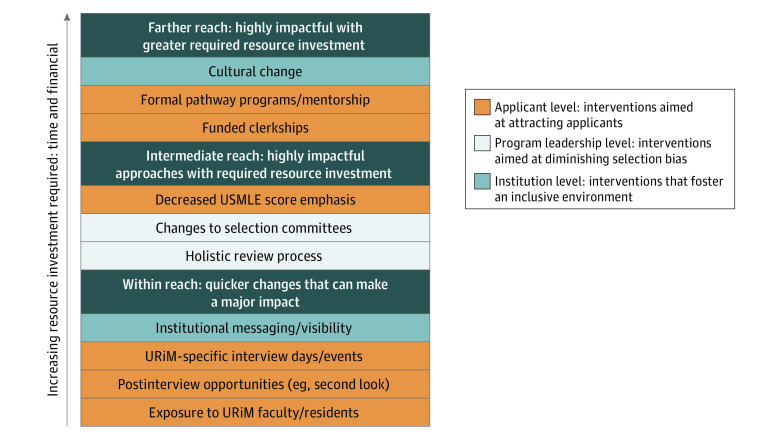
Interventions for Recruitment of Underrepresented in Medicine (URiM) Trainees in Graduate Medical Education USMLE indicates US Medical Licensing Examination.

Multiple diversity measures were described, including those that fall under broad categories of recruitment, residency and fellowship application review, and the interview process. Figure 2 summarizes the intervention components used by the reviewed studies.

### Recruitment

Ten of the 27 studies (37%) described explicit communication regarding institutional commitment to diversity.^9,20,21,23,28,31,33,35,37,39^ This early step entailed making the goal of diversity visible to applicants.^21,31,43^ Some programs attended conferences like the Student National Medical Association to spread the word about their program’s mission.^9,33^ Eight studies reported formal pathway programs as interventions, all of which featured mentorship.^20,21,23,25,35,37,39,45^ One such program was Nth Dimensions,^25^ a long-standing orthopedics summer internship that included lectures, hands-on experiences, research projects, professional development, and ongoing mentorship through subsequent years of medical school. Eight programs leveraged clerkships to increase URiM participation in various specialties.^9,24,26,27,29,39,40,45^ As part of a pilot intervention, Tunson et al^27^ created a 1-month funded visiting emergency medicine rotation that allowed URiM students to rotate in clinical shifts and complete a scholarly project under a faculty mentor’s guidance. A similar program was created for surgery at the University of Pennsylvania, with the goal of providing URiM students exposure to the specialty and connections to set them up for success.^29^

### Application Review

Thirteen programs (48%) deemphasized the US Medical Licensing Examination (USMLE) Step 1 scores when reviewing applications.^23,28,29,30,33,34,36,38,39,40,41,42,44^ Specifically, some programs removed Step 1 minimum scores as a filter for application reviews.^28,30,32^ Thirteen studies (48%) used holistic review, evaluating candidates within the context of their lived experiences and emphasizing their competencies beyond standardized examinations.^23,28,29,30,33,34,36,38,39,40,41,42,44^ Two initiatives used situational awareness tests, placing greater emphasis on applicants’ contributions in their extracurricular activities.^23,34^ Additionally, 2 programs ensured that applications were independently reviewed by several people or by a URiM recruitment committee to diminish unconscious bias against URiM applicants.^27,43^

### Interview Process

Nine programs made intentional efforts to increase exposure of URiM applicants to URiM faculty during interviews.^9,22,23,29,30,37,39,42,43^ Lewis and colleagues^9^ used coffee chats at the end of interview days to connect URiM candidates with URiM faculty members. This served as an opportunity for applicants to inquire about faculty experiences as URiM physicians and available cultural resources in the area. Rymer et al^43^ hosted preinterview dinners and informal receptions where URiM trainees and faculty members could interface with applicants. They also facilitated research meetings and provided materials regarding available resources and opportunities for URiM candidates.^43^ Seven programs created URiM-specific interview days or events.^9,22,23,30,37,43,44^ When possible, URiM applicants were paired with a URiM faculty interviewer.^9,33,43^

### Postinterview Process

Postinterview interventions included sponsoring a URiM-specific Second Look to provide additional opportunities for URiM applicants to meet URiM faculty members and community members.^21,27,37^ Butler et al^29^ leveraged the support of the Alliance of Minority Physicians to conduct targeted outreach to URiM applicants before interviewing, during interview day, and to deliver follow-up communication after interviews. Rymer et al^43^ discussed applicants not only with the general fellowship recruitment committee but specifically with URiM fellows and faculty members.

### Primary Outcomes

Nine studies reported outcomes on URiM applicants,^22,24,25,28,33,34,36,38,43^ with an increase as high as 6.6% from a baseline of 29 URiM of 218 total applicants over a 4-year period.^33^ Eight studies reported outcomes regarding the number of URiM interviewees.^27,28,29,32,33,38,43,44^ Wusu et al^33^ reported the largest increase in URiM interviewees, at 9.9% from a baseline of 15 of 136 interviewees.^33^ Sixteen studies reported outcomes on URiM matriculants, 3 of which^37,39,40^ reported a higher percentage of URiM residents compared with the national average.^21,22,26,27,28,29,30,31,37,39,40,41,42,43,44,45^ Lewis and colleagues^9^ found that increased URiM faculty representation in the residency selection committee resulted in their program’s most racially diverse intern class to date (then, 2019): 6 of 24 categorical residents identified as a minoritized individual. Stoesser and colleagues^44^ reported a 50% URiM-matriculant pool for the 2021 cycle, compared with 0% in 2017. This intern class included 6 Hispanic or Latinx and 2 Southeast Asian residents.^44^

## Discussion

Underrepresentation of Black or African American, Hispanic or Latinx, Native Hawaiian or Pacific Islander, American Indian or Alaska Native, and certain Southeast Asian subgroups persists in medicine, with complex and multifactorial causes signaling the urgent need for widespread solutions. A synthesized analysis of efforts to rectify this issue has been lacking. Our review provides a collection of evidence-based diversity initiatives organized across various stages of the GME recruitment process. As more programs move toward diversity, equity, and inclusion, this compendium of interventions can be used as a starting framework for program directors, trainee selection committees, and GME support staff to organize their own efforts to diversify their programs.

Mentorship programs that allow URiM students to build skills, immerse in clinical experiences, and participate in research opportunities may be effective in increasing the interest and competitiveness of URiM medical students.^35^ Visiting clerkships or even intentionally curated virtual experiences can increase exposure of URiM students to training programs.^46^ While intentional mission statements articulating commitment to diversity, equity, and inclusion are important for URiM students, our review demonstrates that concrete actions to actualize this mission are paramount. Interacting with URiM faculty and trainees can be a powerful avenue to discuss opportunities within the program and address URiM-specific concerns. This effort requires commitment from home departments to recruit and retain URiM faculty and staff.

The use of standardized examination scores in residency application evaluations is well discussed in literature.^47,48,49,50,51,52^ Evidently, USMLE Step 1 score filters disproportionately affect URiM applicants.^53^ Yet, for many specialties, Step 1 scores continue to be an important factor in selecting candidates.^54^ The transition of the Step 1 exam to pass/fail may mitigate the systematic exclusion of URiM applicants in GME recruiting.^55^ However, it can also lead to overreliance on other traditional metrics of evaluation, including school ranking and Alpha Omega Alpha status, both of which have been shown to disadvantage URiM candidates.^56,57,58^ Ultimately, our review found that a holistic approach to GME recruiting was associated with increasing URiM interviewees and matriculants. To maximize the effectiveness of this process, review committee members should interrogate and mitigate their implicit (or explicit) biases.^59^ Committees may also benefit from including a critical mass of URiM faculty, residents, and community members in the review process. Having URiM program leadership may be an important factor in tipping that balance.^6^ Based on findings from Jarman et al,^60^ simply having a diverse faculty and resident body is not enough to guarantee a diverse slate of applicants and matriculants, likely because not all faculty and trainees are involved in the application process. While applying a holistic approach may look different across institutions and individual weighting within holistic approaches is variable, it is critical to give credence to applicants’ lived experiences as a value added to programs.

Fundamentally, a cultural shift is needed in institutions toward prioritizing representation of historically excluded groups in medicine. When diversity and inclusion are explicitly stated institutional goals, they serve as a basis for making concrete changes that drive effective recruitment of URiMs into GME programs. While programs considerably vary in size and culture, dedicated resources serve as a unifying thread. As such, any institution with serious aims of recruiting more URiM trainees must commit time, personnel, and financial resources toward this effort. For example, at one pediatrics residency program, the associate program director received 25% full-time employment (FTE) support for recruitment, along with 30% FTE for the former chief resident. However, faculty working on recruitment spent more than the allocated FTE to create new systems during the first year.^41^ Notably, we did not encounter studies that disclosed the cost with which programs operationalized diversity initiatives. In the setting of multiple potential funding streams, including departmental funds, university allocations, donations, and others, reporting on implementation costs may aid in more widespread operationalization of diversity efforts.

The COVID-19 pandemic drastically reshaped the GME recruiting landscape. In March 2020, medical schools halted clinical rotations for the remainder of the 2019-2020 academic year, necessitating adoption of virtual platforms for all aspects of medical education.^61^ Such a change presented unique challenges to the residency match process, restricting opportunities for away clinical rotations and limiting interviews to virtual formats.^62^ Ngonadi and Barbosa^63^ highlighted barriers faced by URiM applicants in dermatology amidst the pandemic, including greater difficulty in gauging personal fit in programs. Indeed, a study^64^ found that minoritized trainees reported lower fit scores to their top-ranked programs in the setting of COVID-19.

The increasingly virtual nature of GME recruitment necessitates greater attention to programs’ online presence. One study found that out of 8 website elements to illustrate program diversity, general surgery programs included fewer than 3 elements on average.^65^ These elements included nondiscrimination and diversity support statements, demographic characteristics, biographies of residents and faculty with photographs, and resources available to residents. A similar study found that 83% of physical medicine and rehabilitation program websites did not include any of 11 diversity criteria.^66^ Updating websites to reflect program values can be considered easy wins in terms of communicating with URiM applicants. Despite the relative paucity of published data, some programs have shared information regarding their diversity efforts and outcomes in nontraditional spaces such as social media.^67^ Similarly, conference workshops and proceedings may be rich sources of information regarding GME diversity efforts.^68^

Our findings are consistent with those of Mendiola and colleagues,^8^ who sought to elucidate effective strategies and barriers to URiM recruitment in GME. They found that similar strategies were perceived as effective, including showcasing diversity in websites, highlighting diversity and inclusion during interview days, and pairing URiM applicants with URiM faculty. Notably, lack of interest in geographic region, applicant pool diversity, and academic thresholds, among others, were identified as obstacles in diversifying their programs. Further work is necessary to determine interventions aimed at ameliorating these perceived barriers.

### Limitations

This study has limitations. Definitions of URiM are heterogeneous across the reviewed manuscripts. For consistency, we defined URiM using the definition from the Association of American Colleges.^1^ Recognizing that this is an evolving definition, we attempted to be as inclusive as possible when assessing interventions and outcomes. Most studies did not indicate whether any interventions focusing on reaching undergraduate medical students applied only to URiM individuals attending US medical schools vs those in international settings. In addition, most of the included studies used retrospective, observational methods, which limit causal interpretation for the interventions described. Some of the studies did not provide raw data, which limits conclusions regarding the strength of the association between interventions and outcomes assessed. Due to significant differences in size, culture, location, and specialty across the programs, we cannot make conclusions regarding the overall generalizability of the interventions. We encourage reporting of larger sample sizes (perhaps over several recruiting cycles) to facilitate more accurate interpretation of associations of interventions with URiM recruitment, as this will improve generalizability.

Currently published literature may not necessarily encompass the full scope of active interventions to diversify GME. A positive result bias may lead to interventions with lower measured success or those that failed to not be reported. We encourage GME programs to report even these negative studies to advance our collective knowledge regarding effective interventions. Additionally, we noted that only 12 of the studies included outcomes specifying proportion change in URiM trainees at the application, interview, or match and matriculant stages, and only 3 studies reported on proportion change in URiM trainees at all 3 stages. We also encourage programs to report more data on proportion changes in URiM trainees as a result of their initiatives, as this may help similar institutions estimate the potential outcomes of implementing certain interventions when requesting resources to support this work.

There are 9 states in which affirmative action is banned as of 2022, which presents challenges against efforts to specifically address underrepresentation of racially or ethnically minoritized individuals in medicine at programs affiliated with public medical schools.^69^ However, 6 of the published interventions reported in this review took place in states where affirmative action bans are active. Some diversity initiatives may therefore exist whose outcomes are limited to individual institutions and not released to the general medical education community. More published descriptions of active, successful interventions occurring in these states may help other programs navigate these challenges within the legal constraints imposed on them. Additionally, the included studies did not report on outcomes in the setting of the COVID-19 pandemic, which could limit the applicability of some interventions in the current medical education setting. Nevertheless, GME leaders can adapt these existing interventions to fit their specific resources and settings.

## Conclusions

In this scoping review, we provide a summary of interventions at various stages of GME recruiting, which may serve as proposed best practices aimed at diversifying the trainee pool in medical education institutions. Future research is needed to discover how these initiatives change trainee demographic characteristics over longer periods and to understand the full costs associated with operationalizing these interventions.

## Notes

Supplement 1. **eAppendix.** Search Results and Strategies

Supplement 2.
Data Sharing Statement
