# Supplementary material for: Interventions Associated With Racial and Ethnic Diversity in US Graduate Medical Education: A Scoping Review
Source: JAMA Netw Open. 2023 Jan 3;6(1):e2249335. doi: 10.1001/jamanetworkopen.2022.49335 (PMC9856938; doi:10.1001/jamanetworkopen.2022.49335)
Supplement: Supplement 1. — eAppendix. Search Results and Strategies [file jamanetwopen-e2249335-s001.pdf]

Supplemental Online Content

Mabeza RM, Christophers B, Ederaine SA, Glenn EJ, Benton-Slocum ZP, Marcelin JR. Interventions associated with racial and ethnic diversity in US graduate medical education. *JAMA Netw Open.* 2023;6(1):e2249335. doi:10.1001/jamanetworkopen.2022.49335

eAppendix. Search Results and Strategies

This supplemental material has been provided by the authors to give readers additional information about their work.

© 2023 Mabeza RM et al. *JAMA Network Open.*

eAppendix. Search Results and Strategies

Date	Database	Results
2022-01-17	PubMed	582
2022-01-17	Embase	558
2022-01-17	Cochrane	13
2202-01-17	PsycINFO	6
2022-01-17	CINAHL	185
2022-01-17	Scopus	259
2022-01-17	ERIC	12

=1615

2022-01-16 PROSPERO registry search: No similar proto

PubMed (582) *recommend including this PubMed one in the manuscript appendix and not including the others

Database: MEDLINE via PubMed (1946 to present)

(African[tiab] OR black[tiab] OR islander[tiab] OR Latino[tiab] OR latina[tiab] OR latinx[tiab] OR "Native American"[tiab] OR native[tiab] OR indian[tiab] OR indians[tiab] OR asia[tiab] OR Asian[tiab] OR indigenous[tiab] OR hispanic[tiab] OR multiracial[tiab] OR URM[tiab] OR URMS[tiab] OR underrepresented[tiab] OR “under represented”[tiab] OR UIM[tiab] OR “historically excluded”[tiab] OR ethnically[tiab] OR ethnic[tiab] OR minority[tiab] OR minorities[tiab] OR racial[tiab] OR race[tiab] OR racially[tiab] OR cultural[tiab] OR culture[tiab] OR "Racial Groups"[Mesh] OR "Minority Groups"[Mesh] OR "Ethnicity"[Mesh] OR "Cultural Diversity"[mh] OR "Racism"[Mesh])

AND

(diversit*[tiab] OR diverse[tiab] OR diversif*[tiab])

AND

(“residents/fellows”[tiab] OR Intern[tiab] OR interns[tiab] OR internship[tiab] OR internships[tiab] OR residency[tiab] OR residents[tiab] OR resident[tiab] OR residencies[tiab] OR trainee*[tiab] OR “house staff”[tiab] OR “Internship and Residency”[mh] OR "Specialties, Surgical/education"[Mh] OR "Medicine/education"[Mh] OR “Graduate Medical Education”[tiab] OR “surgical training program”[tiab])

© 2023 Mabeza RM et al. *JAMA Network Open.*

AND

("Personnel selection"[mh] OR “Career choice”[mh] OR "Career Mobility"[mh] OR "Vocational Guidance"[Mesh] OR recruit*[tiab] OR encourage*[tiab] OR entrance[tiab] OR preferential*[tiab] OR affirmative[tiab] OR quota*[tiab] OR holistic[tiab] OR applicant*[tiab] OR applications[tiab] OR interviewing[tiab] OR interviewed[tiab] OR interview[tiab] OR admission[tiab] OR “diversity plan”[tiab] OR “diversity plans”[tiab] OR “Selection Bias”[mh] OR “matching program”[tiab] OR match[ti] OR “match process”[tiab] OR NRMP[tiab] OR ERAS[tiab] OR candidates[tiab] OR candidate[tiab])

NOT (“Clinical Study”[PT] OR “Clinical Trial”[PT] OR “Guideline”[PT] OR “Practice Guideline”[PT] OR “Randomized Controlled Trial”[PT])

NOT (animals[mh] NOT (humans[mh] AND animals[mh]))

Limit to English language, published 2011 to present

Embase (558)

((African OR black OR islander OR Latino OR latina OR latinx OR native OR indian OR indians OR Asian OR indigenous OR hispanic OR multiracial OR URM OR UIM OR URMS OR ‘historically excluded’ OR under*represented OR ethnic* OR minority OR minorities OR racial* OR race OR culture* OR cultural*):ti,ab,kw OR 'ethnic group'/exp OR 'minority group'/exp OR 'race'/exp OR 'ancestry group'/exp)

AND

('cultural diversity'/exp OR (diversit* OR diverse OR diversif*):ti,ab,kw)

AND

('medical education'/exp AND (intern:ti,ab,kw OR interns:ti,ab,kw OR internship*:ti,ab,kw OR fellowship*:ti,ab,kw OR 'house staff':ti,ab,kw OR fellow*:ti,ab,kw OR resident:ti,ab,kw OR residents:ti,ab,kw OR residency:ti,ab,kw OR residencies:ti,ab,kw) OR ((medical OR medicine) NEAR/3 (intern OR interns OR internship* OR fellowship* OR 'house staff' OR fellow* OR resident OR residents OR residency OR residencies)) OR 'residency education'/exp OR residency:ti,ab,kw)

AND

(recruiti* OR holistic* OR applicant* OR application* OR interview* OR match* OR 'NRMP' OR candidacy* OR cadidate*):ti,ab,kw

OR

© 2023 Mabeza RM et al. *JAMA Network Open.*

((Match* OR divers*) NEAR/4 (plan OR plans OR planning OR program* OR effort* OR manage* OR process* OR evaluat*))

OR

'organization and management'/exp OR 'personnel management'/exp OR 'program evaluation'/exp OR 'selection bias'/exp

NOT ('note'/it OR 'letter'/it OR 'editorial'/it)

Limit to English

Cochrane (13)

Search Name: Residents diversity -- Russyan

Date Run: 17/01/2022 05:09:15

Comment: more specific terms for residency

ID Search Hits

#1 MeSH descriptor: [Ethnic Groups] explode all trees 4668

#2 MeSH descriptor: [Cultural Diversity] explode all trees 75

#3 (African OR black OR islander OR Latino OR latina OR latinx OR "Native American" OR native OR indian OR indians OR asia OR Asian OR indigenous OR hispanic OR multiracial OR URM OR URMS OR underrepresented OR “under represented” OR UIM OR ethnically OR ethnic OR minority OR minorities OR racial OR race OR racially OR cultural OR culture):ti,ab,kw (Word variations have been searched) 105096

#4 (diversi* OR diverse):ti,ab,kw (Word variations have been searched) 12483

#5 MeSH descriptor: [Minority Groups] explode all trees 383

#6 #1 OR #2 OR #3 OR #5 105215

#7 MeSH descriptor: [Specialties, Surgical] explode all trees and with qualifier(s): [education - ED] 640

#8 MeSH descriptor: [Internship and Residency] explode all trees 1335

#9 MeSH descriptor: [Education, Medical, Graduate] explode all trees 1527

#10 (internship OR internships OR residency OR residencies OR fellowship OR felllowships OR "house staff"):ti,ab,kw 3089

#11 #7 OR #8 OR #9 OR #10 3620

#12 #4 AND #6 AND #11 21

#13 (recruit* OR encourage* OR entrance OR preferential* OR affirmative OR quota* OR holistic OR applicant* OR applications OR interviewing OR interviewed OR interview OR admission OR “diversity plan” OR “diversity plans” OR “matching program” OR “match process” OR NRMP OR candidates OR candidate):ti,ab,kw (Word variations have been searched) 255729

#14 (match):ti (Word variations have been searched) 2354

#15 MeSH descriptor: [Personnel Selection] explode all trees 54

#16 MeSH descriptor: [Career Choice] explode all trees 66

#17 #13 OR #14 OR #15 OR #16 257808

#18 #12 AND #17 13

(1 review, 12 trials)

PsychINFO (6)

S6 S1 AND S2 AND S3 AND S4 Limiters - Published Date: 20130101-20211231; English

Search modes - Boolean/Phrase Interface - EBSCOhost Research Databases

Search Screen - Advanced Search

Database - APA PsycInfo 6

S5 S1 AND S2 AND S3 AND S4 Search modes - Boolean/Phrase Interface - EBSCOhost Research Databases

Search Screen - Advanced Search

Database - APA PsycInfo 11

S4 ((((DE "Interviews" OR DE "Job Applicant Interviews") OR (DE "Professional Development" OR DE "Professional Certification" OR DE "Professional Competence" OR DE "Professional Ethics" OR DE "Professional Licensing" OR DE "Professional Recognition" OR DE "Professional Socialization" OR DE "Professional Specialization")) OR (DE "Occupational Guidance")) OR (DE "Career Development" OR DE "Career Change" OR DE "Personnel Placement" OR DE "Personnel Promotion")) OR (DE "Program Evaluation" OR DE "Educational Program Evaluation" OR DE "Mental Health Program Evaluation") OR TI (recruit* OR encourage* OR entrance OR preferential* OR affirmative OR quota* OR holistic OR applicant* OR applications OR interviewing OR

© 2023 Mabeza RM et al. *JAMA Network Open.*

interviewed OR interview OR admission OR “diversity plan” OR “diversity plans” OR “matching program” OR “match process” OR NRMP OR candidates OR candidate) OR AB (recruit* OR encourage* OR entrance OR preferential* OR affirmative OR quota* OR holistic OR applicant* OR applications OR interviewing OR interviewed OR interview OR admission OR “diversity plan” OR “diversity plans” OR “matching program” OR “match process” OR NRMP OR candidates OR candidate) Search modes - Boolean/Phrase Interface - EBSCOhost Research Databases

Search Screen - Advanced Search

Database - APA PsycInfo 854,474

S3 DE "Racial and Ethnic Groups" OR DE "African Cultural Groups" OR DE "Arabs" OR DE "Asians" OR DE "Blacks" OR DE "European Cultural Groups" OR DE "Indigenous Populations" OR DE "Latinos/Latinas" OR DE "Romanies" OR DE "Tribes" OR DE "Whites" OR DE "Affirmative Action" OR DE “Racism” OR DE "Minority Groups" Search modes - Boolean/Phrase Interface - EBSCOhost Research Databases

Search Screen - Advanced Search

Database - APA PsycInfo 135,026

S2 (DE "Diversity" OR DE "Cultural Diversity" OR DE "Diversity in the Workplace" OR DE "Ethnic Diversity" OR DE "Diversity in the Workplace") OR TI diverse OR TI Diversity Search modes - Boolean/Phrase Interface - EBSCOhost Research Databases

Search Screen - Advanced Search

Database - APA PsycInfo 25,511

S1 (DE "Medical Residency") OR TI residency OR TI resident OR AB residency OR TI fellows OR TI fellow OR KW residency OR (graduate NEAR/2 medical) Search modes - Boolean/Phrase Interface - EBSCOhost Research Databases

Search Screen - Advanced Search

Database - APA PsycInfo 16,409

CINAHL (185)

S19 S10 AND S15 Limiters - Published Date: 20110101-20221231

Narrow by SubjectGeographic: - usa

Narrow by Language: - english

Search modes - Boolean/Phrase Interface - EBSCOhost Research Databases

© 2023 Mabeza RM et al. *JAMA Network Open.*

Search Screen - Advanced Search

Database - CINAHL 185

S18 S10 AND S15 Limiters - Published Date: 20110101-20221231

Narrow by Language: - english

Search modes - Boolean/Phrase Interface - EBSCOhost Research Databases

Search Screen - Advanced Search

Database - CINAHL 313

S17 S10 AND S15 Narrow by Language: - english

Search modes - Boolean/Phrase Interface - EBSCOhost Research Databases

Search Screen - Advanced Search

Database - CINAHL 394

S16 S10 AND S15 Search modes - Boolean/Phrase Interface - EBSCOhost Research Databases

Search Screen - Advanced Search

Database - CINAHL 398

S15 S11 OR S12 OR S13 OR S14 Search modes - Boolean/Phrase Interface - EBSCOhost Research Databases

Search Screen - Advanced Search

Database - CINAHL 743,983

S14 TI (recruit* OR encourage* OR entrance OR preferential* OR affirmative OR quota* OR holistic OR applicant* OR applications OR interviewing OR interviewed OR interview OR admission OR “diversity plan” OR “diversity plans” OR “Selection Bias”[mh] OR “matching program” OR match[ti] OR “match process” OR NRMP OR ERAS OR candidates OR candidate) OR AB (recruit* OR encourage* OR entrance OR preferential* OR affirmative OR quota* OR holistic OR applicant* OR applications OR interviewing OR interviewed OR interview OR admission OR “diversity plan” OR “diversity plans” OR “Selection Bias”[mh] OR “matching program” OR match[ti] OR “match process” OR NRMP OR ERAS OR candidates OR candidate) Search modes - Boolean/Phrase Interface - EBSCOhost Research Databases

Search Screen - Advanced Search

Database - CINAHL 699,951

© 2023 Mabeza RM et al. *JAMA Network Open.*

S13 (MH "Vocational Guidance") OR (MH "Vocational Education") Search modes - Boolean/Phrase Interface - EBSCOhost Research Databases

Search Screen - Advanced Search

Database - CINAHL 1,620

S12 (MH "Personnel Selection+") OR (MH "Personnel Retention") Search modes - Boolean/Phrase Interface - EBSCOhost Research Databases

Search Screen - Advanced Search

Database - CINAHL 19,737

S11 (MH "Career Planning and Development") OR (MH "Career Mobility+") OR (MH "Clinical Ladder") OR (MH "Vocational Guidance") Search modes - Boolean/Phrase Interface - EBSCOhost Research Databases

Search Screen - Advanced Search

Database - CINAHL 34,801

S10 S7 AND S8 AND S9 Search modes - Boolean/Phrase Interface - EBSCOhost Research Databases

Search Screen - Advanced Search

Database - CINAHL 1,038

S9 S1 OR S5 Search modes - Boolean/Phrase Interface - EBSCOhost Research Databases

Search Screen - Advanced Search

Database - CINAHL 98,579

S8 S2 OR S3 OR S4 OR S6 Search modes - Boolean/Phrase Interface - EBSCOhost Research Databases

Search Screen - Advanced Search

Database - CINAHL 455,152

S7 TI (diversit* OR diverse OR diversif*) OR AB (diversit* OR diverse OR diversif*) Search modes - Boolean/Phrase Interface - EBSCOhost Research Databases

Search Screen - Advanced Search

Database - CINAHL 79,720

S6 TI (African OR black OR islander OR Latino OR latina OR latinx OR "Native American" OR native OR indian OR indians OR asia OR Asian OR indigenous OR hispanic OR multiracial OR URM OR URMS OR underrepresented OR “under

© 2023 Mabeza RM et al. *JAMA Network Open.*

represented” OR UIM OR “historically excluded” OR ethnically OR ethnic OR minority OR minorities OR racial OR race OR racially OR cultural OR culture) OR AB (African OR black OR islander OR Latino OR latina OR latinx OR "Native American" OR native OR indian OR indians OR asia OR Asian OR indigenous OR hispanic OR multiracial OR URM OR URMS OR underrepresented OR “under represented” OR UIM OR “historically excluded” OR ethnically OR ethnic OR minority OR minorities OR racial OR race OR racially OR cultural OR culture) Search modes - Boolean/Phrase Interface - EBSCOhost Research Databases

Search Screen - Advanced Search

Database - CINAHL 384,724

S5 TI (“residents/fellows” OR Intern OR interns OR internship OR internships OR residency OR residents OR resident OR residencies OR trainee* OR “house staff” OR “Graduate Medical Education” OR “surgical training program”) OR AB (“residents/fellows” OR Intern OR interns OR internship OR internships OR residency OR residents OR resident OR residencies OR trainee* OR “house staff” OR “Graduate Medical Education” OR “surgical training program”) Search modes - Boolean/Phrase Interface - EBSCOhost Research Databases

Search Screen - Advanced Search

Database - CINAHL 89,273

S4 (MH "Ethnic Groups+") Search modes - Boolean/Phrase Interface - EBSCOhost Research Databases

Search Screen - Advanced Search

Database - CINAHL 156,968

S3 (MH "Racism") Search modes - Boolean/Phrase Interface - EBSCOhost Research Databases

Search Screen - Advanced Search

Database - CINAHL 9,271

S2 (MH "Cultural Diversity") Search modes - Boolean/Phrase Interface - EBSCOhost Research Databases

Search Screen - Advanced Search

Database - CINAHL 14,746

S1 (MH "Internship and Residency") OR (MH "Interns and Residents") Search modes - Boolean/Phrase Interface - EBSCOhost Research Databases

Search Screen - Advanced Search

© 2023 Mabeza RM et al. *JAMA Network Open.*

Database - CINAHL 27,494

Scopus (259)

TITLE-ABS-KEY (diversi*) AND TITLE-ABS-KEY (african OR black OR islander OR latino OR latina OR latinx OR "native american" OR native OR indian OR indians OR asia OR asian OR indigenous OR hispanic OR multiracial OR urm OR urms OR underrepresented OR "under represented" OR uim OR "historically underrepresented" ethnically OR ethnic OR minority OR minorities OR racial OR race OR racially OR cultural OR culture OR "racism" OR "racial group*" OR "minority group*" OR "ethnicity") AND TITLE-ABS-KEY ("residents/fellows" OR intern OR interns OR internship OR internships OR residency OR residencies OR trainee* OR "house staff" OR "graduate medical education" OR "surgical training program") AND (TITLE-ABS-KEY (personnel OR career OR recruiting OR recruitment OR encourage* OR entrance OR preferential* OR affirmative OR quota* OR holistic OR applicant* OR applications OR interviewing OR interviewed OR interview OR admission OR "diversity plan" OR "diversity plans" OR bias OR nrmp OR candidates OR candidate OR "match process") OR TITLE ((match OR matching))) AND (LIMIT-TO (PUBYEAR , 2022) OR LIMIT-TO (PUBYEAR , 2021) OR LIMIT-TO (PUBYEAR , 2020) OR LIMIT-TO (PUBYEAR , 2019) OR LIMIT-TO (PUBYEAR , 2018) OR LIMIT-TO (PUBYEAR , 2017) OR LIMIT-TO (PUBYEAR , 2016) OR LIMIT-TO (PUBYEAR , 2015) OR LIMIT-TO (PUBYEAR , 2014) OR LIMIT-TO (PUBYEAR , 2013) OR LIMIT-TO (PUBYEAR , 2012) OR LIMIT-TO (PUBYEAR , 2011)) AND (EXCLUDE (DOCTYPE , "le") OR EXCLUDE (DOCTYPE , "no") OR EXCLUDE (DOCTYPE , "ch") OR EXCLUDE (DOCTYPE , "ed") OR EXCLUDE (DOCTYPE , "er")) AND (LIMIT-TO (LANGUAGE , "English"))

ERIC (16)

Monday, January 17, 2022 11:30:59 PM

Query Limiters/Expanders Last Run Via Results

S9 S3 AND S6 AND S7 AND S8 Search modes - Boolean/Phrase Interface - EBSCOhost Research Databases

Search Screen - Advanced Search

Database - ERIC 16

S8 (((DE "Career Awareness") OR (DE "Professional Development")) AND (DE "Personnel Directors" OR DE "Personnel Evaluation" OR DE "Personnel Integration" OR DE "Personnel Management" OR DE "Personnel Needs")) OR (DE "Recruitment"

© 2023 Mabeza RM et al. *JAMA Network Open.*

OR DE "Faculty Recruitment" OR DE "Student Recruitment" OR DE "Teacher Recruitment") OR TI ((recruit* OR encourage* OR entrance OR preferential* OR affirmative OR quota* OR holistic OR applicant* OR applications OR interviewing OR interviewed OR interview OR admission OR “diversity plan” OR “diversity plans” OR “matching program” OR “match process” OR NRMP OR candidates OR candidate)) OR AB ((recruit* OR encourage* OR entrance OR preferential* OR affirmative OR quota* OR holistic OR applicant* OR applications OR interviewing OR interviewed OR interview OR admission OR “diversity plan” OR “diversity plans” OR “matching program” OR “match process” OR NRMP OR candidates OR candidate)) Search modes - Boolean/Phrase Interface - EBSCOhost Research Databases

Search Screen - Advanced Search

Database - ERIC 297,556

S7 ((DE "Racial Bias") OR (DE "Ethnic Groups" OR DE "Alaska Natives" OR DE "American Indians" OR DE "Anglo Americans" OR DE "Canada Natives" OR DE "Chinese Americans" OR DE "Eskimos" OR DE "Filipino Americans" OR DE "French Canadians" OR DE "Hawaiians" OR DE "Hmong People" OR DE "Italian Americans" OR DE "Japanese Americans" OR DE "Korean Americans" OR DE "Mexican Americans" OR DE "Pacific Islanders" OR DE "Polish Americans" OR DE "Samoan Americans" OR DE "Spanish Americans")) OR (DE "Ethnic Groups") OR TI racial OR AB racial OR TI Ethnic* OR AB ethnic* Search modes - Boolean/Phrase Interface - EBSCOhost Research Databases

Search Screen - Advanced Search

Database - ERIC 82,556

S6 S4 OR S5 Search modes - Boolean/Phrase Interface - EBSCOhost Research Databases

Search Screen - Advanced Search

Database - ERIC 64,327

S5 TI diverse OR TI Diversity OR TI diversify OR AB diverse OR AB Diversity OR AB diversify Search modes - Boolean/Phrase Interface - EBSCOhost Research Databases

Search Screen - Advanced Search

Database - ERIC 63,604

S4 DE "Diversity (Faculty)" OR DE "Diversity (Institutional)" Search modes - Boolean/Phrase Interface - EBSCOhost Research Databases

Search Screen - Advanced Search

Database - ERIC 2,567

© 2023 Mabeza RM et al. *JAMA Network Open.*

S3 S1 OR S2 Search modes - Boolean/Phrase Interface - EBSCOhost Research Databases

Search Screen - Advanced Search

Database - ERIC 4,526

S2 OR TI residency OR TI resident OR AB residency OR TI fellows OR TI fellow OR KW residency OR ((graduate OR resident*) NEAR/2 medical) Search modes - Boolean/Phrase Interface - EBSCOhost Research Databases

Search Screen - Advanced Search

Database - ERIC 3,952

S1 DE "Graduate Medical Education"Search modes - Boolean/Phrase Interface - EBSCOhost Research Databases

Search Screen - Advanced Search

Database - ERIC 1,308

© 2023 Mabeza RM et al. *JAMA Network Open.*
