# Supplementary material for: Interventions Associated With Racial and Ethnic Diversity in US Graduate Medical Education: A Scoping Review
Source: JAMA Netw Open. 2023 Jan 3;6(1):e2249335. doi: 10.1001/jamanetworkopen.2022.49335 (PMC9856938; doi:10.1001/jamanetworkopen.2022.49335)
Supplement: Supplement 2. — Data Sharing Statement [file jamanetwopen-e2249335-s002.pdf]

Data Sharing Statement

Mabeza. Interventions Associated With Racial and Ethnic Diversity in US Graduate Medical Education. *JAMA Netw Open*. Published January 03, 2023.
doi:10.1001/jamanetworkopen.2022.49335

Data
Data available: No

Additional Information
Explanation for why data not available: Given the nature of the study, all data utilized are available as previously published materials.
